# Evaluation of U.S. Army Soldiers wearing a back exosuit during a field training exercise

**DOI:** 10.1017/wtc.2023.16

**Published:** 2023-07-07

**Authors:** P. R. Slaughter, K. M. Rodzak, S. J. Fine, C. C. Ice, D. N. Wolf, K. E. Zelik

**Affiliations:** 1 Department of Mechanical Engineering, Vanderbilt University, Nashville, Tennessee, United States; 2 Department of Biomedical Engineering, Vanderbilt University, Nashville, Tennessee, United States; 3 Department of Physical Medicine & Rehabilitation, Vanderbilt University, Nashville, Tennessee, United States

**Keywords:** exosuits, performance augmentation, industry, design

## Abstract

Back overuse injuries are a significant problem in the U.S. Army, responsible for nearly a quarter of musculoskeletal injuries. Back exosuits are wearable devices that relieve musculoskeletal strain, make lifting easier, and could potentially reduce Soldier overuse injuries. But published studies have not evaluated exosuits during realistic field operations to assess acceptability to Soldiers. We tested a back exosuit on field artillery Soldiers during a field training exercise. Afterward, Soldiers completed a survey to quantify their satisfaction, intent to use, and performance impact of the exosuit. Feedback was overwhelmingly positive: Approximately 90% of Soldiers reported that exosuits increased their ability to perform their duties, and 100% said that if the exosuit were further developed and made available to them, they would be likely to wear it. These numerical survey results indicated that exosuits can provide a practical and acceptable way to assist lifting and augment physical performance during realistic Army operations without interfering with other duties.

## 1. Introduction

Back overuse injuries are a significant problem in the U.S. Army and are responsible for nearly a quarter of all musculoskeletal injuries to Soldiers (U.S. Army Public Health Center, 2019). Overuse injuries result from repetitive or prolonged musculoskeletal loading, such as from heavy lifting and other strenuous jobs performed by Soldiers. An average of 167,926 back overuse injuries were diagnosed in the Army each year between 2016 and 2019, which equates to 460 Soldier back overuse injuries every day and one back overuse injury diagnosed every 3.1 minutes (U.S. Army Public Health Center, 2016; 2017; 2018; 2019). Low back injuries result in 1.2–1.7 million limited or lost duty days for Soldiers each year (Roy et al., 2022). Back injuries are also the most common condition factoring into disability-related discharges from the Army (Walter Reed Army Institute of Research, 2019).

Occupational back *exos*, including rigid exoskeletons and soft exosuits, are wearable devices that relieve musculoskeletal strain and could reduce Soldier overuse injuries. Many back exos relieve the user’s muscles and spine during lifting and bending by providing an extension moment about the lumbar spine (Howard et al., 2020; Kermavnar et al., 2020; Bär et al., 2021; Lamers and Zelik, 2021). Back exos are currently used in various civilian industries (e.g., logistics, manufacturing, agriculture, and construction) and are gaining interest to enhance human safety when traditional ergonomic controls are not practical or feasible (Nussbaum et al., 2019; Zelik et al., 2022). Back exos could be adapted to fit and support Soldiers (Crowell et al., 2019; Farris et al., 2022; Proud et al., 2022), many of whom routinely perform strenuous lifting, loading, and bending work.

The effectiveness of back exos to reduce back strain has been confirmed through a large body of evidence comprised of computational modeling, biomechanics and ergonomics laboratory experiments, and field studies. For instance, studies have repeatedly shown that back exos reduce muscle strain and spinal compression forces, which are risk factors for low back pain and overuse injury (Kermavnar et al., 2020; Lamers and Zelik, 2021). Back exos have also been observed to reduce muscle fatigue (Lamers et al., 2020; dos Anjos et al., 2022) and metabolic rate (Baltrusch et al., 2019; Alemi et al., 2022; Schmalz et al., 2022) during bending and lifting tasks, which could enhance endurance or other performance outcomes during physical tasks. Much of the scientific evidence on exos has been generated over the last decade in laboratory experiments or controlled field studies (Kermavnar et al., 2020; Crea et al., 2021). Based on these advances in knowledge, the critical questions about exos are no longer related to whether exos can reduce musculoskeletal loading or assist movement. Instead, pressing questions are now associated with factors that underlie user acceptance and organizational adoption, including comfort, usability, freedom of movement, and impacts during real work (i.e., not just lifting but also during other job tasks) (Omoniyi et al., 2020; Elprama et al., 2022). As with any tool, ergonomic intervention, or wearable device, users derive no benefits if they are not willing or interested in using an exo, highlighting the importance of evaluating user experiences and perceptions.

Critical unanswered questions include: will back exos be satisfactory (acceptable) to Soldiers, will Soldiers be interested in wearing exos during their duties, and will exos affect Soldier performance during real (i.e., full-speed, unconstrained) operations? These questions about acceptability, future use intent, and real-world performance impact are particularly pertinent to the U.S. Army, given various prior exo technologies that were not acceptable to Soldiers nor adopted by the Army (Keller, 2021). Prior exo prototypes relieved musculoskeletal demands and enhanced Soldier performance (for a specific subset of tasks during controlled experiments); however, these devices ultimately failed to meet other Soldier needs or perform adequately during realistic field use. Previous barriers to Soldier acceptance and Army adoption include devices being too heavy, bulky, rigid, expensive, power-consuming, unreliable, operationally complex, or interfering with other job tasks (Cornwall, 2015; Scharre et al., 2018; Crowell et al., 2019). Relatedly, a prevalent takeaway from recent studies is that an exo may assist with one task and reduce injury risk but interfere with other movements, duties, or environments (Spada et al., 2018; Baltrusch et al., 2019; Hensel and Keil, 2019; Omoniyi et al., 2020). Collectively, the history of Army exos and these commonly observed challenges related to movement interference motivate the need to evaluate exos during realistic field use to understand the actual exo impact and user experience.

Although the U.S. Army has trialed various back exos (e.g., in laboratory evaluations, Soldier touch points, demonstrations), there is need for more extensive testing with Soldiers (Mudie et al., 2018; Crowell et al., 2019). To date, no published scientific studies have evaluated how Soldiers perceive exos during actual, full-speed operations. The objective of this study was to fill this knowledge gap in the scientific literature by evaluating a back exo worn by field artillery Soldiers during a field training exercise.

### 1.1. Motivation and project background

For context, this study was conducted as part of the Army Pathfinder Program, a Congressional initiative to advance the U.S. Army’s modernization goals and support the innovation of Soldier-inspired, research-based technologies. This program works by partnering Soldiers with academic researchers and engineers, alongside other technology translation experts and military partners, to develop Soldier-driven solutions to Soldier-identified problems. Our research team at Vanderbilt University (Nashville, TN) collaborated with the 101^st^ Airborne Division (Ft. Campbell, KY) on this project. Soldiers in the 101^st^ identified strength and endurance limitations during the field artillery mission as a significant source of overburdening and fatigue which can negatively impact their health, performance, and readiness. These Soldiers routinely lift, load, unload, and handle heavy equipment and ammunition, often weighing 15–60 kg or more (e.g., for group lifts) such as when emplacing (setting up) or displacing (taking down) a Howitzer (long-range artillery weapon).

Over 12 months, Vanderbilt and the 101st engaged in focus groups, interviews, observations, and a series of design sprints that involved testing and feedback from over 100 Soldiers. During this design period, we began with a passive-elastic back exosuit technology that our team at Vanderbilt had previously researched and developed (Lamers et al., 2018, 2020; Yandell et al., 2022), and transitioned to civilian commercial use (HeroWear, 2020). We then redesigned the exosuit into a prototype that integrates with Soldiers’ gear (e.g., body armor) and meets other Soldier needs. The design of the prototype, called the Soldier Assistive Bionic Exosuit for Resupply (SABER), is summarized below, but the focus of this article is on the field study evaluation. After the design period, we tested the SABER prototypes on Soldiers during one of their scheduled multi-week field trainings, in which Soldiers conducted field training exercises. These exercises are the pinnacle of training for field artillery Soldiers who are not actively deployed to practice missions in a realistic combat environment.

## 2. Methods

### 2.1. Participants

Eleven Soldiers (nine males and two females, aged 19–36) from the 101st volunteered to wear an exosuit prototype during a portion of their field training exercises. The Military Occupational Specialties (MOS) of these Soldiers were as follows: five 13B (Cannon Crewmembers), one 91B (Wheeled Vehicle Mechanic), one 91C (Utilities Equipment Repairer), one 91D (Tactical Power Generation Specialist), one 92F (Petroleum Supply Specialist), and two 92G (Culinary Specialists). All Soldiers participated in Howitzer firing and resupply mission tasks as part of an M119 live fire exercise. Only one of the 11 Soldiers had previously worn an exosuit (i.e., an earlier prototype during the SABER iterative design period). All participants gave written, informed consent before testing. To avoid undue influence, commanding officers were not involved in or present during study recruitment, consent, introduction, or surveys. The protocol was approved by the Vanderbilt University Institutional Review Board and the U.S. Army Human Research Protections Office.

### 2.2. Device overview

**Figure 1.**
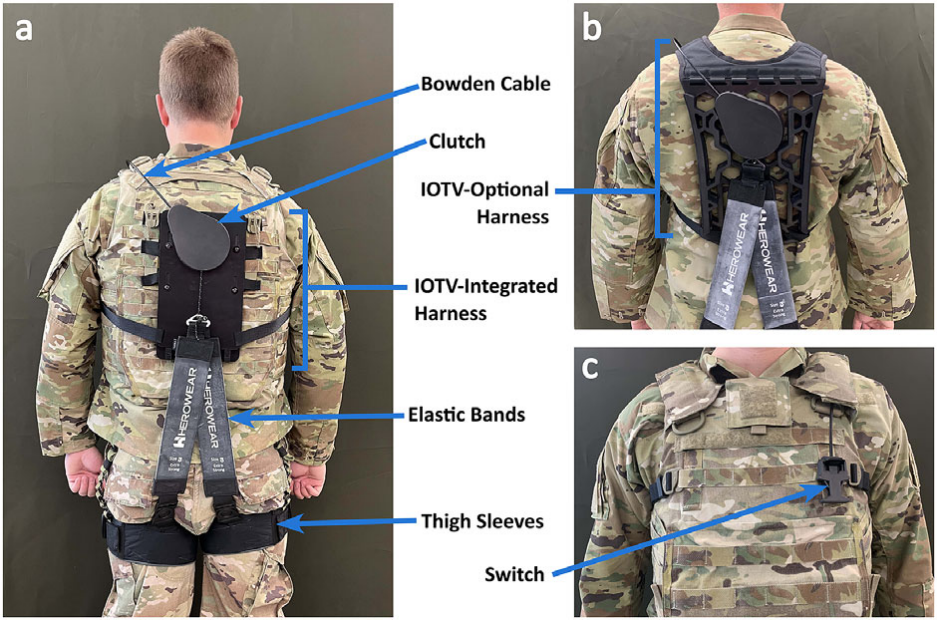
Overview of SABER prototypes. Shown are the (a) back view of the exosuit with the IOTV-Integrated harness, (b) back view with IOTV-Optional Harness, and (c) front view showing the switch. Key exosuit components are labeled. In panel (b), the IOTV is not being worn so that IOTV-Optional harness is visible; however, during field testing, an IOTV was worn over the top.

Each Soldier wore a SABER prototype (Figure 1). SABER is an unpowered back exosuit containing no motors or batteries. SABER assists biomechanically using elastic bands as detailed in our prior exosuit publications (Lamers et al., 2018, 2020). SABER engages/disengages assistance similar to the HeroWear Apex (HeroWear, 2020), but SABER was completely redesigned to integrate with a Soldier’s standard issue gear. The exosuit consists of a harness (upper-body interface), thigh sleeves (lower-body interface), clutch-switch system (for turning exosuit assistance on/off), and elastic bands (configured along the back to act as an artificial set of back and hip extensor muscles). Elastic bands used were commercial-off-the-shelf bands purchased from HeroWear. SABER prototypes were designed to be compatible with these modular elastic bands for ease of sizing. All Soldiers wore strength S2000 (extra strong) bands, except for one who wore strength S1500 (strong) based on band availability in their size. The entire SABER prototype weighed 1.2 kg (2.7 pounds).

A user engages exosuit assistance by pulling down on a switch located on the left shoulder, which causes the clutch (located on the upper back) to lock. Then, when the user bends forward, the elastic bands along the back stretch, creating an assistive torque about the lumbar spine and reducing strain on the back muscles and discs (Lamers et al., 2018; Goršič et al., 2021). The switch connects to the clutch via a Bowden cable transmission. A user utilizes the engaged mode during lifting and bending tasks to relieve back strain. A user disengages assistance by pinching the same switch on the left shoulder; this causes the clutch to unlock, so another weaker spring (located inside the clutch) is now in series with the elastic bands. The weaker spring is part of a retractable spring mechanism (similar to a key chain retractor or retractable dog leash), which allows the user to have a free range of motion (i.e., to move or bend against negligible resistance from the weaker retractable spring). Users utilize the disengaged mode during non-lifting tasks such as running, sitting in a vehicle, or climbing a ladder.

We developed two SABER upper-body harness styles for Soldiers. The IOTV-Integrated harness (Figure 1a) attaches to the outside (backside) of the Improved Outer Tactical Vest (IOTV) that Soldiers wear. The IOTV-Optional harness (Figure 1b) fits underneath the IOTV or can be worn without an IOTV. These harness styles emerged from the iterative design process, and both were viable solutions for integrating with the Soldier gear. Functionally, the exosuit operates (e.g., on/off switch) the same way with both harness styles. Both harness styles also use the same elastic bands, and thus provide similar levels of back assistance. Since all Soldiers were required to wear an IOTV during field training exercises, we treated these two configurations (IOTV-Integrated and IOTV-Optional, Figure 1) as equivalent interventions for this field study. Six Soldiers wore an IOTV-Integrated harness, and five Soldiers wore an IOTV-Optional harness during testing, based on prototypes available. For data analysis and reporting purposes, we considered Soldiers wearing either harness to be wearing a SABER prototype; and we drew no distinction between them.

### 2.3. Device fitting, training, and acclimation

Due to the ongoing field training exercise, the time to fit, train, and acclimate to the SABER prototype was limited to about 10–20 min per Soldier. There was only a short break in the exercise during which we could recruit, consent, fit, and train Soldiers before they had to get back to work. Soldiers were fit and trained as best as possible by our research team. We instructed Soldiers that toggling the switch on provides help when lifting and bending, and toggling the switch off turns that assistance off. We then gave Soldiers a short time to practice using the exosuit. Complete acclimation was not possible due to the nature of this field training exercise and the need for Soldiers to respond rapidly to mission orders.

### 2.4. Field Training Exercise

**Figure 2.**
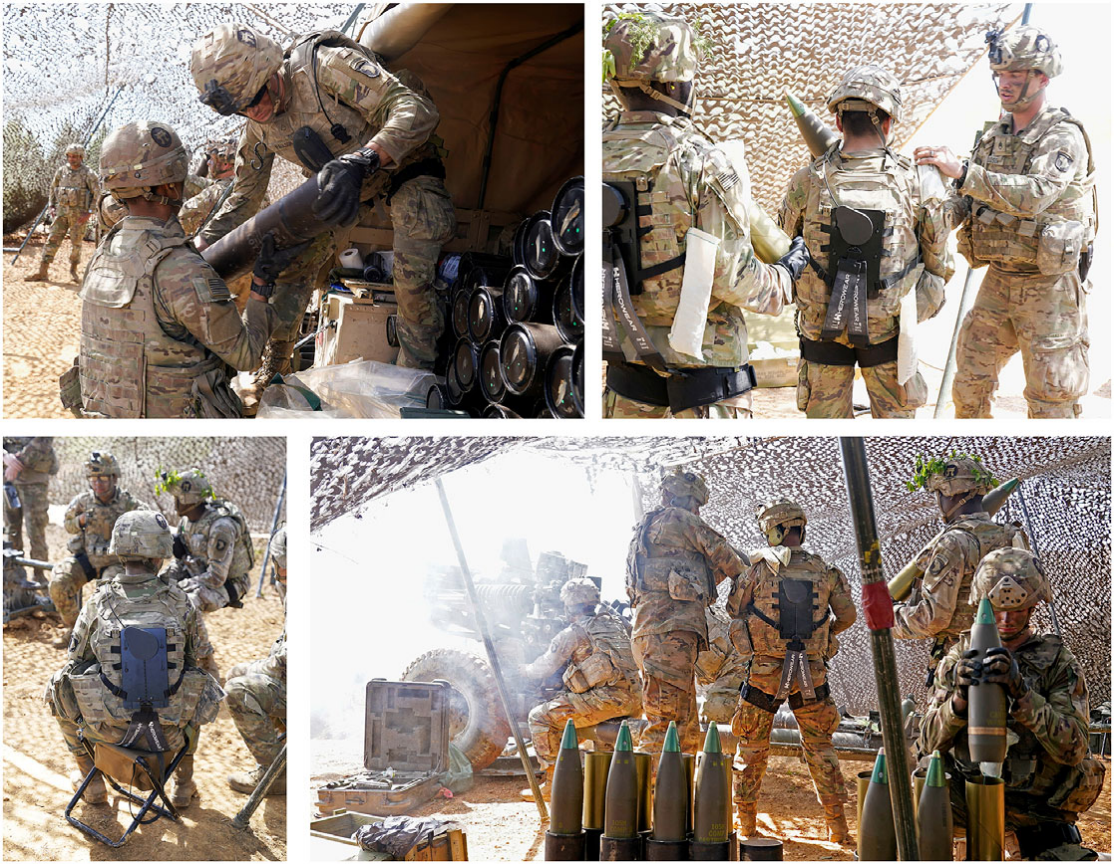
Photos of Soldiers wearing exosuits in the field training exercise depict representative duties during firing and resupply portions of the mission. Top-left: Soldier unloads Howitzer rounds from the back of a vehicle. Top-right: Soldiers carry Howitzer rounds and charges (white propellant bags). Bottom-left: Soldier sits during a brief break in the action. Bottom-right: Soldiers prepare, carry, and deliver rounds to be fired during the exercise.

Each Soldier wore a SABER prototype continuously for 2 to 4 hr during the firing and resupply portion of the field training exercise. At the time of testing, Soldiers had already been living in the field for over 2 weeks. On the day of testing, exosuits were donned by Soldiers approximately 10 hr into a 14-hr artillery mission, which began at midnight. During this time, over eight hundred 105 mm Howitzer rounds (about 18 kg or 40 pounds each) were lifted, loaded, and fired. The Soldiers were stationed across six M119 Howitzer crews and involved in ammunition preparation, loading, resupply, and firing duties; this was a highly dynamic environment in which each Soldier performed various movements and tasks (Figure 2). Most of the Soldiers wearing exosuits worked as cannon crewmembers. One of their primary tasks was to repeatedly lift and carry rounds to a Howitzer about 20 feet away. The Soldiers also performed tasks such as climbing in and out of vehicles, unloading equipment out of confined spaces, running, jumping, sitting, and kneeling on the ground. It was up to each Soldier to determine when to engage/disengage the exosuit assistance.

### 2.5. Data collection and analysis

After the exercise, each Soldier completed a written survey. The survey was a seven-point Likert-scale evaluation adapted from one previously created by the U.S. Army to evaluate exos for Soldiers (survey shared in private communication from U.S. Army DEVCOM Soldier Center). The goals of the survey were to quantify Soldiers’ self-reported satisfaction (acceptance), intent to use, and performance impact of the exosuit.

Satisfaction was evaluated by rating, on a scale from *Very Dissatisfied* to *Very Satisfied*, attributes of the exosuit that are important to user experience and acceptance, specifically: lifting assistance, ease of donning, ease of doffing, ease of use, snag hazards, range of motion, heat retention, physical comfort, fit, weight, and overall performance. Rating an attribute as *Neutral* (4 of 7 on the scale) signified that it was okay, not eliciting either a positive or negative response.

Performance impact was evaluated by rating if Soldiers felt the exosuit increased or decreased their ability to perform their tasks on a scale from *Great Decrease* to *Great Increase.* The neutral rating (4 of 7 on the scale) was *No Impact.* Alternatively, Soldiers could select *Not Sure* if they felt the time duration or job tasks performed during testing were insufficient to assess the effect of the exosuit on their performance.

Use intent was evaluated by Soldiers rating if they would be likely to wear this exosuit for their job if it were developed into a product and made available to them, on a scale from *Very Unlikely* to *Very Likely.* The neutral rating (4 of 7) on the scale was entitled *Neither Unlikely Nor Likely*, for Soldiers who were unsure if they would want to wear an exosuit in the future but were not opposed to this possibility.

Numerical survey results were compiled, analyzed, and plotted. The survey included space for brief written responses to explain ratings. In addition, each Soldier also provided brief verbal feedback (about 1–3 min in duration) to our research team. This was not a structured interview, and due to time constraints, Soldiers were not all asked exactly the same questions. However, most Soldiers were asked “Can you describe your experience wearing the suit?”. We also asked “If we continue to develop this kind of suit and make it available to Soldiers, is it something you would be interested in using?”. Some responses were useful for corroborating or explaining numerical survey results. We, therefore, included a few Soldier quotes in the Discussion section as supplementary information and to further inform interpretation of results.

## 3. Results

**Figure 3.**
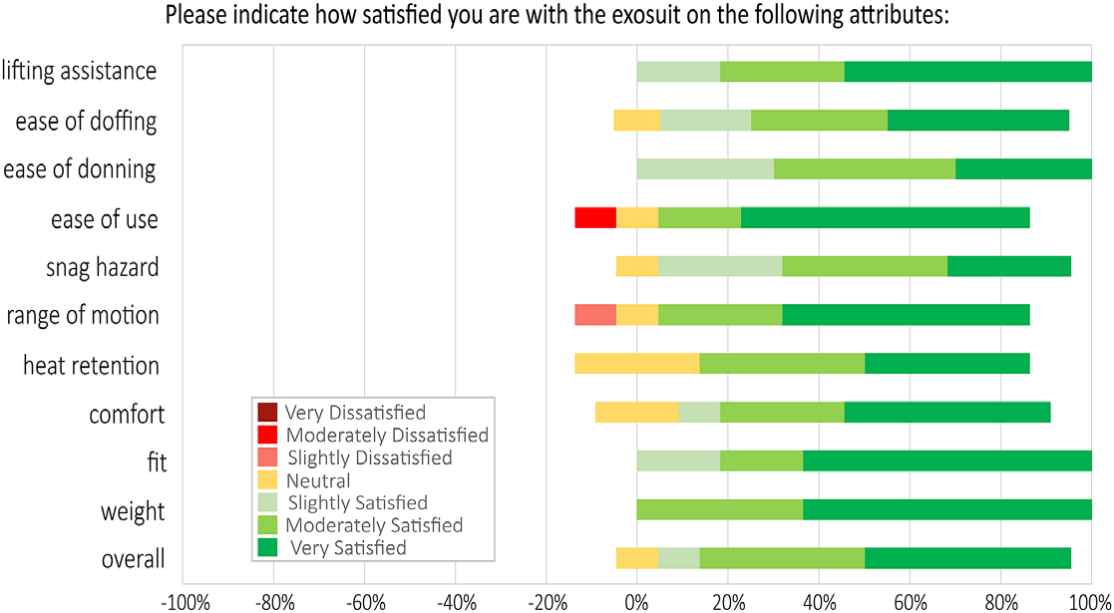
Soldier satisfaction with exosuit attributes, based on Likert-scale surveys. Soldier ratings were generally positive, with about 90% of responses being satisfied (slightly, moderately, or very), 8% of responses being neutral, and < 2% of responses being dissatisfied (slightly or moderately) with individual attributes. X-axis percentages are provided for reference to easily visualize positive (satisfied) responses to the right versus negative (dissatisfied) responses to the left of 0%.

Soldiers were satisfied with the attributes of the SABER prototype (Figure 3). For instance, 100% of Soldiers were satisfied (slightly, moderately, or very) with the weight, fit, ease of donning, and lifting assistance provided by the SABER prototype. Also, 91% of Soldiers (10 of 11) were satisfied overall, while 9% (1 of 11) reported neutral satisfaction. Out of 121 survey responses (11 Soldiers *x* 11 attributes), Soldiers only reported two dissatisfaction ratings—one for range of motion and one for ease of use—representing <2% of responses.

**Figure 4.**
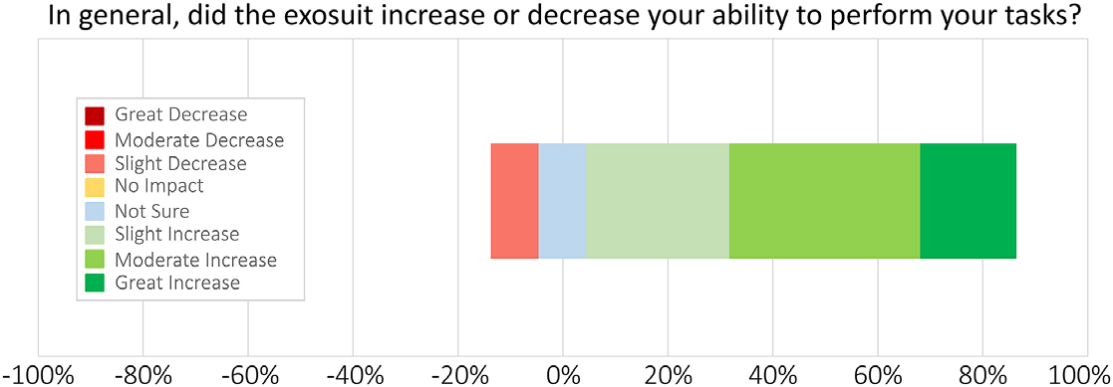
Exosuit effects on Soldier performance, based on Likert-scale surveys. 90% of Soldiers responded that the exosuit slightly, moderately, or greatly increased their ability to perform their tasks, excluding one *Not Sure* respondent (see Discussion for more details on this Soldier). X-axis percentages are provided for reference to easily visualize positive (increase) responses to the right versus negative (decrease) responses to the left of 0%.

**Figure 5.**
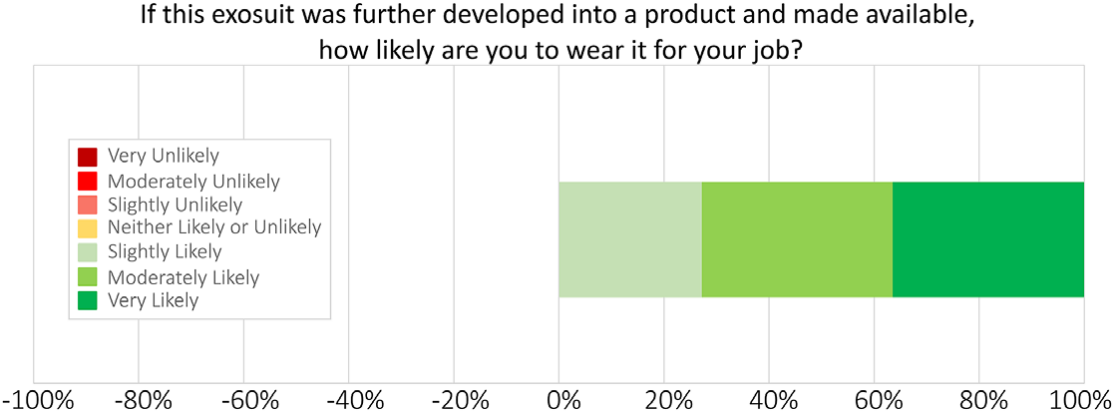
Soldiers’ interest in using exosuits for their job, based on Likert-scale surveys. 100% of Soldiers responded that if this exosuit was further developed into a product and made available, they would be slightly, moderately, or very likely to wear it for their job. X-axis percentages are provided for reference to easily visualize positive (likely) responses to the right versus negative (unlikely) responses to the left of 0%.

Soldier ratings on the SABER prototype were positive regarding performance benefits and interest in using the exosuit on the job (Figures 4 and 5). 90% of Soldiers (nine of 10, excluding one *Not Sure* response) reported the exosuit slightly, moderately, or greatly increased their ability to perform their field artillery tasks (Figure 4). One Soldier marked *Not Sure* on this question because, during the exosuit testing period, he was placed into a job role that did not involve lifting; thus, we excluded him from this percentage calculation. 100% of Soldiers (11 of 11) reported that they would be slightly, moderately, or very likely to wear this back exosuit for their job if it were developed into a product and made available to them (Figure 5).

## 4. Discussion

Survey results from Soldiers using the SABER prototypes during the field training exercise were overwhelmingly positive. Three key takeaways from this study were as follows:

1. This back exosuit was satisfactory (acceptable) to Soldiers during short-term field testing. The exosuit did not cause discomfort. The exosuit could be integrated with a Soldier’s existing gear and could be used in realistic operational environments without interfering with other tasks, gear or equipment.
2. Soldiers felt that this type of exosuit increased their ability to perform their duties during critical and dynamic field operations by making lifting easier.
3. If this back exosuit were further developed and made available, then Soldiers expressed that they would be likely to wear it on the job.

### 4.1. Insights from Soldier feedback on wearing the exosuit

Verbal Soldier feedback aided in interpretation of the numerical survey results (Figures 3–5) by providing additional context and specificity. For instance, Soldier comments gave insight into how and where they felt lifting assistance; an attribute which they rated favorably (Figure 3). In terms of specific tasks, multiple Soldiers mentioned the exosuit was helpful for lifting, loading, and unloading artillery rounds, with one Soldier explaining the exosuit “helped greatly when picking up rounds and moving from A to B”. In terms of where on the body they felt assisted, Soldiers shared a few perspectives. Feedback included “it definitely helped my legs,” “a lot of support, especially on the back,” and “it corrected my posture.” Feedback also gave insight into why most Soldiers rated the exosuit as easy to use (Figure 3). Soldiers discussed the ability to switch exosuit assistance on and off, with one Soldier explaining “I was easily able to engage quickly when I knew I needed to use it, and just disengage when I was just running around back and forth.” While these quotes give a sense of how Soldiers perceived the exosuit during field operations, it is important to note that this feedback is based on short-term usage (2–4 hr). See the Limitations sections below for an extended discussion of this topic and future considerations.

We also identified a few areas for improvement based on negative Soldier survey responses and verbal feedback. These were mostly from one-off events or circumstances. We believe these were not fundamental issues with the exosuit attributes or functionality but rather opportunities for improvements in device design, fitting, and training.

One Soldier expressed slight dissatisfaction (Figure 3) because he felt his range of motion when bending downwards was limited. Given the stature of this Soldier and this feedback, it is likely that he needed a lower stiffness elastic band or other fit adjustments, but due to time constraints in this field study, these were not feasible. In practice, this could be addressed through an exosuit fitting process in which a Soldier self-selects their preferred elastic band stiffness (rather than being assigned), along with exosuit fit checks that are performed during the acclimation process.

One Soldier reported that the exosuit slightly decreased their ability to perform tasks (Figure 4); however, it turns out this was referring to their ability to quickly doff the exosuit during a bathroom break. The Soldier explained, “I went to use the restroom – it’s kind of hard to take it off,” and therefore rated the exosuit’s ease of use as moderately unsatisfactory (Figure 3). We attribute this negative experience to insufficient training time, as it was unnecessary for them to doff the entire exosuit. With more training time, we would have instructed users on a simple way to disconnect a portion of the exosuit, which quickly and easily allows them to use the bathroom, likely avoiding this negative experience. Of note, in other parts of the survey, this same Soldier reported they were *Very Satisfied* with the exosuit’s assistance, and they would be *Very Likely* to use the exosuit for their job, indicating that their overall experience wearing the exosuit was positive.

A few Soldiers mentioned minor snag incidents; however, none resulted in any negative ratings on the surveys (Figure 3). All snags reported involved the Howitzer charges (small bags of propellant connected by string) getting caught on parts of the exosuit. Soldiers sling charges (white bags visible in the top-right photo of Figure 2) over their shoulders when carrying a round to the Howitzer. Two Soldiers reported that the string on the charges snagged on the exosuit’s switch (near the left shoulder), one said that the string snagged on the Bowden cable, and one said that the string snagged on the back of the IOTV-Integrated harness. In all situations, snags were minor and quickly resolved such that Soldiers were able to complete their duties. In the future, slight alterations to the exosuit design, such as tacking down the Bowden cable or refining the shape of the switch, could further reduce snag risks. Overall, the survey results indicate the snag hazards were acceptable to Soldiers (Figure 3), but individual comments nonetheless identify minor areas for design improvements.

### 4.2. Broader applications

**Figure 6.**
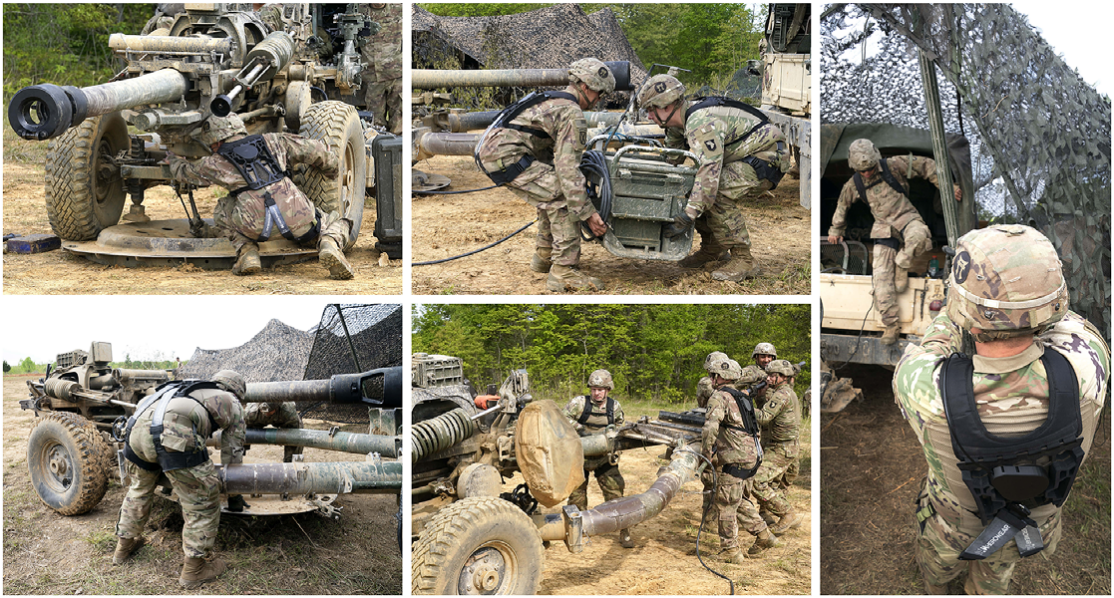
Photos of Soldiers wearing exosuits during a Howitzer emplacement and displacement exercise, as part of an *N* = 2 case study exploring additional field artillery duties which supplement the formal live fire exercise. Top-left: Soldier reaches under the Howitzer during displacement. Top-middle: Two Soldiers lift and carry a communications box (77 kg). Bottom-left: Soldiers lift a firing platform (102 kg) during Howitzer emplacement. Bottom-middle: Soldiers lift the Howitzer trail end as part of emplacement. Right: One Soldier climbs out of the back of the vehicle while another secures a netting pole.

Exosuits could also benefit other parts of the M119 field artillery mission beyond firing and resupply. A smaller case study opportunity (*N* = 2) arose for further testing of Soldiers wearing exosuits during simulated Howitzer emplacement and displacement (Figure 6). Afterward, Soldiers completed the same surveys as outlined in Methods. The results were similarly positive. Both Soldiers reported via the survey a slight performance increase. One Soldier also reported he would be moderately likely to wear the exosuit on the job, and the other reported slightly likely. These Soldiers were generally satisfied with the SABER attributes and did not rate any attributes of the exosuit as dissatisfactory.

Other Army jobs and MOSs (Military Occupational Specialties) could also benefit from musculoskeletal relief provided by back exosuits. This could include Soldiers in other field artillery (e.g., M777, M109) and distribution units. These units also perform heavy repeated lifts as a regular part of their duties. During the iterative design period of this study, we completed informal exosuit testing where Soldiers from these other units provided preliminary but positive feedback, similar to what is presented in the Results (Figures 3–5). Various other sustainment, logistics, and maintenance jobs may also benefit from exosuits.

The quantitative survey results and verbal Soldier feedback on SABER support the conclusion that this kind of exosuit could potentially help reduce back overuse injuries, pain, and lost duty time. More broadly, exos could help a wide range of jobs in the Army, as well as in other Defense branches and various industries in the civilian sector. See the Appendix for a brief overview of frequently asked questions relevant to how and where exos might complement Army occupational health programs and technological advances.

### 4.3. Study limitations

This study focused on self-reported outcomes after short-term exo use. Because exos are wearable devices that exert physical forces on the user, it was not feasible to blind users from the intervention. In addition, the participants were aware that our research team was involved in the development of the exo prototype tested. There is some risk that this knowledge could influence the participants’ perceptions or their willingness to provide negative feedback. However, in our experience working with over 100 Soldiers during iterative development and informal testing, we found Soldiers to be very candid and they frequently provided negative feedback. We do not have any reason to believe or expect the Soldiers were any less candid in this field study than they had been previously.

Various other factors affect long-term user acceptance, organizational adoption, and how user perceptions change over time (Hensel and Keil, 2019; Ferreira et al., 2020). Future studies should aim to quantitatively measure the effect of exos on Soldier performance, operational metrics, and injury risk, while also monitoring for any potential unintended effects. Existing ergonomic assessment tools predict that back exos will reduce musculoskeletal disorder risk factors (e.g., Di Natali et al., 2021; Zelik et al., 2022; van der Have et al., 2023). But we expect that a large number of users will need to wear exos long-term to assess the impact on injury reduction empirically.

We only studied one type of exo (an unpowered back exosuit). Still, we anticipate opportunities and niches for a wide variety of exos (e.g., passive, powered, back, neck, shoulder, leg) to provide physical augmentation and relief to Soldiers. However, in our experience, commercial-off-the-shelf exos developed for civilians will be limited in their military use cases. These exos will generally need to be adapted or redesigned for military use (Proud et al., 2022), particularly in the field, to meet Soldiers’ needs and integrate well into their gear and environment. We believe it is crucial to include users (Soldiers) early on and throughout the exo development or adaptation process.

As discussed above, there was limited time for Soldier fitting, training, and acclimation. Poggensee and Collins (2021) highlighted the benefits of sufficient (and risks of insufficient) exo training. This study found that benefits from wearing an ankle exoskeleton increased 2–3x after extended training and acclimation compared to when users initially used the device. In civilian industries, it is routine for workers to acclimate to a new exo over days or weeks of use, which is at least 100x longer than the acclimation permitted in our field study; this was one of the drawbacks but realities to evaluation during a field training exercise. We expect Soldiers would further benefit from SABER with time and learning, as with any new tool. Whenever possible, we would advise more extended training and acclimation periods to understand assistive benefits to the user best. Nevertheless, the Soldier feedback and results from this field study were encouraging despite limited training with the exosuit and other limitations acknowledged for this field study.

## 5. Conclusions

Survey responses from Soldiers on wearing SABER, an unpowered back exosuit prototype, during a field training exercise were generally positive. Soldiers perceived this exosuit as positively impacting their performance. Soldiers also reported that they would likely wear this back exosuit for their job if it were further developed and made available to them. This study demonstrates that this kind of elastic, mode-switching back exosuit can provide a practical and acceptable way to reduce back strain and make lifting easier for Soldiers in the field during dynamic and realistic operations without interfering with other job tasks or gear.

## Data Availability

Data presented will be provided by the corresponding author upon request.

## Appendix

Here we briefly address a few frequently asked questions about exo science and the potential role of exo technology in the military.

### Where do exos fit amongst Army ergonomics & technology advances?

Exos complement existing ergonomic controls and can be part of a comprehensive occupational health program. Safety professionals should continue to prioritize ergonomic design and controls, but often these are not feasible, particularly during military operations in the field. It is evident from Army injury data (U.S. Army Public Health Center, 2016; 2017; 2018; 2019; Roy et al., 2022) and Soldier feedback that Soldiers are overburdened. Thus, the Army should explore new tools and technologies that can help relieve this burden on Soldiers. Exos are one new tool that can reduce musculoskeletal strain and injury risk. While some civilian organizations have elected to classify exos as personal protective equipment (Butler and Gillette, 2019), others consider exos engineering controls or tools. It is yet to be seen how the U.S. Army will classify and implement exos.

Exos also complement advances in automation. Automation such as autonomous resupply is being developed by/for the Army, but only to help with a few selected tasks along the artillery supply chain from “factory to fire.” In the civilian sector, increased automation has been observed to increase physical demands on certain workers along the supply chain due to higher throughput (Evans, 2020). Thus, we expect there will continue to be heavy—and potentially elevated—physical demands on Soldiers as Army technology and automation advances, including in critical, remote, and confined places where automation is impractical.

### How are back exos different from back belts?

Exos overcome the limitations of back belts, which have not proven effective at preventing back injuries. Posture aids such as back belts were previously trialed in occupational environments; however, studies have generally not found them to reduce back strain (Thomas et al., 1999), fatigue (Majkowski et al., 1998), or injuries (Bigos et al., 2009; Verbeek et al., 2011; Sowah et al., 2018). Consequently, the National Institute for Occupational Safety and Health does not recommend using back belts to prevent workplace injuries (National Institute for Occupational Safety and Health, 1994). Relevant to the military, a joint services working group found “moderate to strong evidence that back belts/supports are ineffective,” which “supports the Department of Defense position that back support belts are not personal protective equipment, and use of these devices for the prevention of back injuries is not endorsed” (Bullock et al., 2010). Compared to back belts, exos are a fundamentally and functionally different class of wearable device, which reduce musculoskeletal loads using mechanical leverage and therefore show promise in reducing overuse injury risks. For instance, automotive manufacturers have reported that exos for the shoulders have reduced workplace injuries (Barrero, 2019) and medical visits (Kim et al., 2022) in longitudinal studies, and published ergonomic models indicate that back exos are projected to reduce low back injuries (e.g., Zelik et al., 2022). However, larger-scale longitudinal validation studies are still needed.

### What types of exos might benefit Soldiers?

Exos are wearable tools designed to solve specific problems and, as a result, come in many shapes, sizes, and varieties. Although most prior exos developed for the Army have been rigid, robotic devices, one takeaway from this study is that exos do not need to be rigid, motorized, or overly complex to benefit Soldiers. The key is developing and matching the right exo (wearable tool) to the right job. The structure of the device (rigid versus soft) and actuation (powered versus passive) are design choices, not user requirements. End-user needs should inform these design choices. For instance, SABER is made primarily of soft materials (e.g., textiles, elastomers) and uses passive-elastic actuation to assist lifting while containing no motor or batteries. The SABER design reflects its purpose and approach: It was designed in close collaboration with its eventual end-users (Soldiers) to assist specific parts of the body during specific physical tasks while minimizing form factor, weight, complexity, and movement interference. There are vast opportunities for researchers and developers to collaborate with other end-users (Soldiers and civilians) to develop exos that address specific pain points without needing to augment the entirety of a user’s physical performance.
